# Compartmentalized Biomolecular Condensates via Controlled Nucleation

**DOI:** 10.34133/research.0505

**Published:** 2024-10-17

**Authors:** Chong Wang, Linyi Zhang, Luoran Shang

**Affiliations:** Shanghai Xuhui Central Hospital, Zhongshan-Xuhui Hospital, and the Shanghai Key Laboratory of Medical Epigenetics, the International Co-laboratory of Medical Epigenetics and Metabolism (Ministry of Science and Technology), Institutes of Biomedical Sciences, Fudan University, Shanghai, China.

## Abstract

This commentary underscores the importance and implications of the study “Biomolecular condensates with complex architectures via controlled nucleation,” led by Jan C. M. van Hest and Tuomas P. J. Knowles, published in *Nature Chemical Engineering*. The research team developed a novel system to investigate the structure of biological condensates using quaternized amylose, carboxymethylated amylose, and single-stranded DNA. They successfully created multiphase droplets with distinct dense phases and demonstrated that droplet architecture can be controlled through temperature and salt concentration adjustments. This study offers valuable insights into the formation and function of membraneless organelles in cells and suggests promising applications for designing biomimetic materials and therapeutic strategies.

Biomolecular condensation has been identified as a crucial process for creating specialized dynamic compartments within cells, termed membraneless organelles (MLOs). MLOs are dynamic biomolecular condensates typically consisting of specific proteins and nucleic acids (RNA and/or DNA) while lacking lipid membranes. Many MLO-composing proteins contain intrinsically disordered regions [1]. By undergoing phase separation into liquid-like or gel-like phases, MLOs facilitate the compartmentalization of specific biological functions within cells. They can promote biochemical reactions through simultaneously concentrating substrates and enzymes or suppress reactions by sequestering some factors away from the action site [2]. Biomolecular condensation is crucial in many cellular processes such as gene expression regulation, stress response, and signal transduction, and it is also implicated in diseases like cancer and neurodegenerative disorders [3–6]. This involvement underscores the importance of biomolecular condensation in maintaining cellular function and highlights their potential as therapeutic targets, thus leading to the development of therapeutics targeting biomolecular condensates. Despite their widespread occurrence, many aspects of biomolecular condensate formation and their broader impact on cellular processes remain poorly understood. Understanding the physicochemical principles underlying biomolecular condensate functionality requires detailed studies conducted both in vivo and in vitro. However, because of the challenges in controlling conditions for the liquid–liquid phase separation process, a reliable model for the study of the structure and dynamic changes of biomolecular condensates is highly expected.

Complex coacervates show great potential as models for biomolecular condensates. Because complex coacervation can be driven by similar electrostatic interactions, the formed coacervates can simulate some of the properties of biomolecular condensates such as hierarchical organization and sequestration ability [7]. In recent years, studies on coacervates have achieved remarkable progress, driven by the demand for simple model systems enabling systematic and quantitative investigation of MLO characteristics [8–12]. Coacervates can be generated rapidly by mixing 2 components, typically of opposite charges, and this results in the formation of simple microdroplets [7]. Although being convenient models in some aspects, simple droplets fall short of replicating the subcompartmentalized architecture of many biological condensates within cells. Deciphering the contributions of the mesoscale structure of biological condensates to cellular processes and disease development can pave the way for innovative therapeutic strategies and biomimetic materials design. However, controlling the multicompartmental structure of coacervate droplets has been less explored.

Aiming to explore the mesoscale structural features of biological condensates, Erkamp et al. proposed a multiphase complex coacervation system, as shown in Fig. 1 [13]. In this study, the authors introduce a multiphase coacervate model system and shed light on the thermodynamic principles that drive the nucleation of condensates. This approach offers a way to control the architecture of multiphase condensates at the mesoscale, opening up new possibilities for their functionality and applications. Writing in *Nature Chemical Engineering*, they utilized an aqueous solution of quaternized amylose (Q-amylose, positively charged), carboxymethylated amylose (Cm-amylose, negatively charged), and single-stranded DNA (negatively charged) to generate multiphase separated droplets out of a dilute phase (Phase 1) [13]. Because the density of negative charges on DNA is higher than that on Cm-amylose, 2 dense phases formed within the droplets: a DNA-poor one (Phase 2) and a DNA-rich one (Phase 3). Q-amylose and DNA were most concentrated in Phase 3, whereas Cm-amylose was most abundant in Phase 2 (Fig. 2A).

**Fig. 1.**
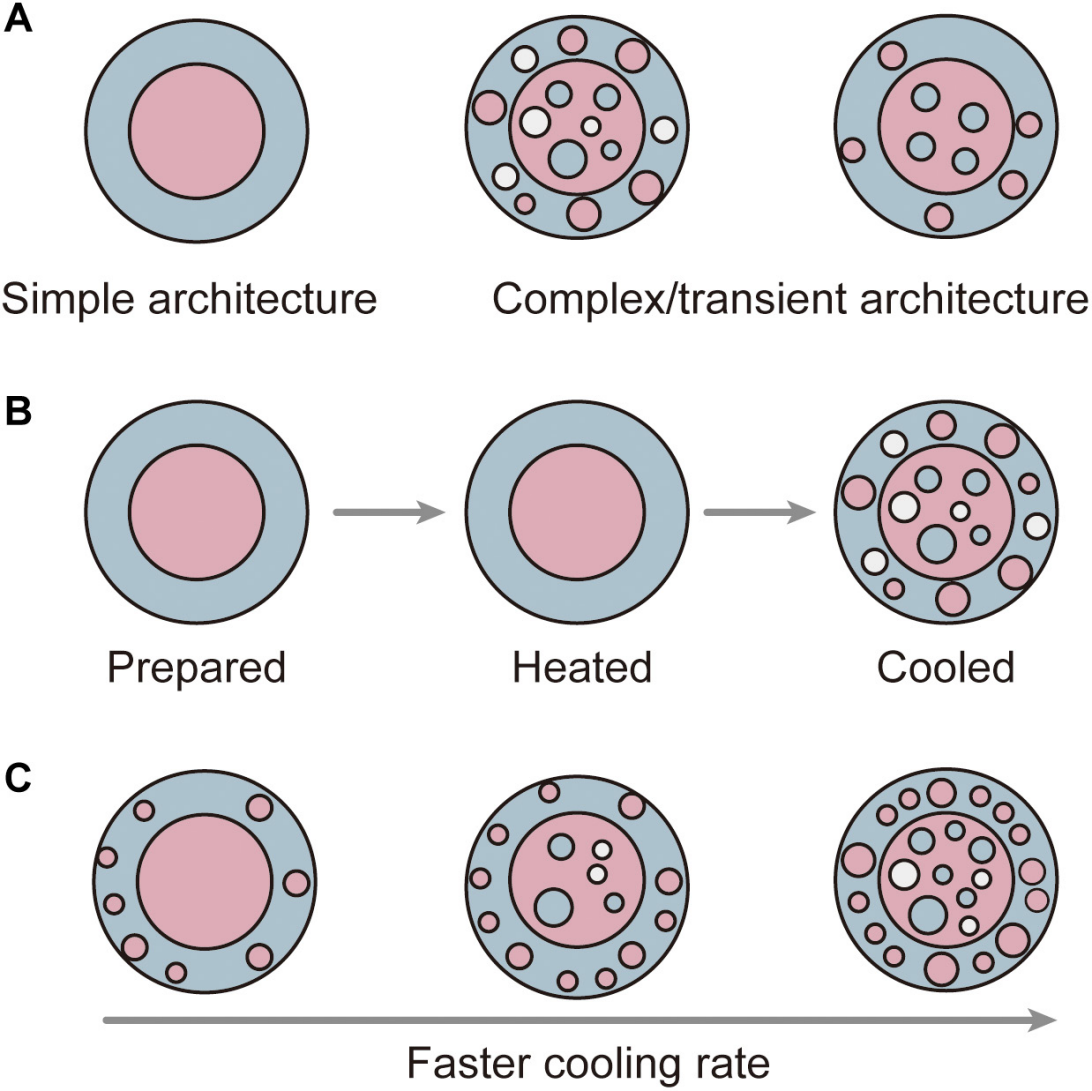
Schematics of condensate architectures, multiphase droplets, and their complexity with cooling rate. (A) Schematic showing the various architectures of the condensates. (B) Schematic showing the generation of multiphase condensate droplets. (C) Schematic showing the complexity of multiphase condensate droplets is positively correlated to the cooling rate.

**Fig. 2.**
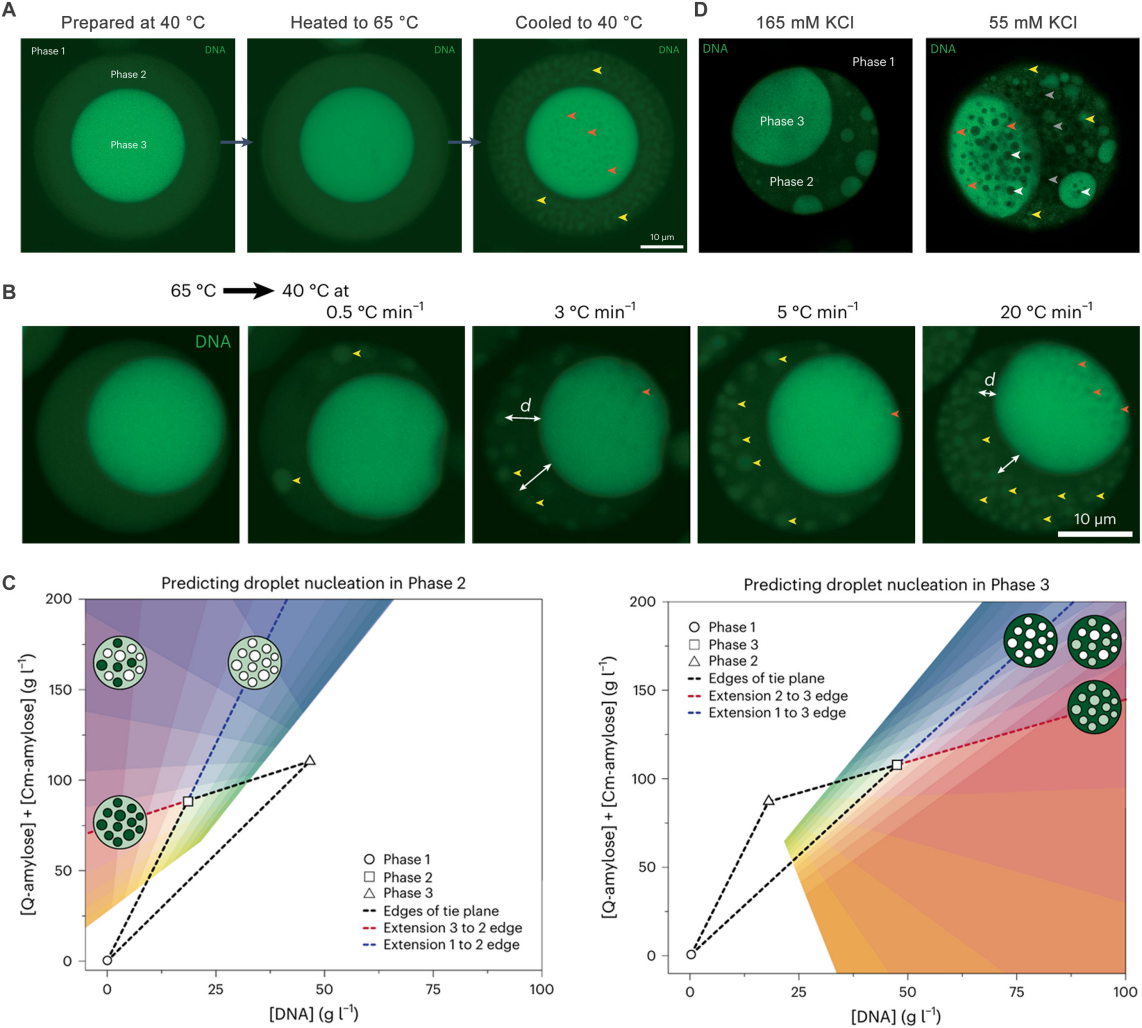
Formation and behavior of condensate droplets under varying temperature, cooling rate, and salt concentration. (A) Heating the condensates to 65 °C and then cooling it to 40 °C forms droplets of Phase 2 in Phase 3 (orange) and Phase 3 in Phase 2 (yellow). (B) As the cooling progressed more rapidly, more condensate droplets were formed at closer distances. (C) Look-up diagrams for predicting nucleation or mixing of different phases based on mass balance. (D) Lowering the salt concentration causes the formation of more complex condensate architectures.

Prepared at 40 °C, Phase 1 surrounded the condensates, with Phase 2 partly engulfing Phase 3. When the condensates were heated to 65 °C and then rapidly cooled to 40 °C, Phase 3 droplets nucleated within Phase 2 and vice versa (Fig. 2A). During cooling, the amount of DNA in Phase 1 remained relatively constant, but the increased hydrophobic interactions reduced DNA concentration in Phase 2 and increase it in Phase 3. Additionally, with fluorescent recovery after photobleaching experiments, it was found that diffusion of molecules was slow in the condensate system. Therefore, it was challenging for the condensates to alter their composition upon changes of environmental conditions. However, nucleating can rapidly adjust the system’s composition by generating new droplets, thereby bypassing the slow diffusion process and enabling a quicker response to environmental changes. Accordingly, the number of nucleated droplets formed was found to be positively correlated to the cooling rate (Fig. 2B).

To design specific condensate architectures, the authors used phase diagrams to analyze and predict the composition in different phases under varying experimental conditions, based on mass balance (Fig. 2C). Additionally, the authors adjusted temperature and salt concentration to regulate electrostatic interactions, a key factor in the coacervation process, thereby altering the composition of different phases and driving the system out of equilibrium. These changes, in turn, triggered nucleation, markedly impacting the system’s overall architecture (Fig. 2D). They also showed that coacervates with complex architectures and large interface areas could quickly absorb cargo into Phase 3. This finding suggests that by tuning the architecture of these complex coacervates, it is possible to control the exchange dynamics at the interfaces, which offers new potential for designing drug delivery systems. Overall, by leveraging restricted diffusion within dense condensates and triggering composition changes through rapid alterations in experimental conditions, the authors created complex architectures in a multiphase condensate model. They also used phase diagrams and tie planes to elucidate the formation of complex architectures and further demonstrated the potential applications of these intricate structures. Based on this, further studies may be conducted to analyze complex coacervation as well as biological condensates formed by other components, as MLOs in vivo contain a multitude of components. Furthermore, apart from the temperature and salt concentration changes investigated in this article, living cells can regulate electrostatic interactions through processes such as phosphorylation and dephosphorylation. Therefore, investigating the effects of phosphorylation or dephosphorylation on complex MLO structures via in vitro models could have important biomedical implications.

In summary, this study offered insight into how complex condensate structures arise out of equilibrium, contributing to the understanding of the dynamic behavior and structural variety of MLOs. It also highlighted the potential applications of adjusted multiphase condensate systems and helped to enhance our understanding of MLO structure and function. As highlighted in many similar studies, it is possible to alter the equilibrium state of coacervate droplets with complex structures by tweaking factors such as surface tension, polarity, and interaction strength between the various components within multiphase coacervate droplets. However, current research is still far from replicating the phase separation environments found in living organisms, which are regulated by various biochemical processes occurring at mild conditions. Despite its limitations, this study’s approach to analyzing and predicting phase diagrams under various nonequilibrium conditions could greatly advance research on intracellular phase-separated organelles, especially in multilayered biological condensates like the nucleolus. The presence of complex condensate architectures will allow researchers to incorporate compartmentalization-related functions into condensates, paving the way for diverse applications in biotechnology, artificial cell engineering, and studies on the origin of life.
